# Supplementary material for: Association between oral microbiome and seven types of cancers in East Asian population: a two-sample Mendelian randomization analysis
Source: Front Mol Biosci. 2023 Nov 21;10:1327893. doi: 10.3389/fmolb.2023.1327893 (PMC10702768; doi:10.3389/fmolb.2023.1327893)
Supplement: Supplementary file 1 [file DataSheet1.ZIP › Supplementary Materials/MR plots of saliva/saliva═╝/ovarian cancer/pheno.2209_to_ovarian cancer_leave_one_out.pdf]

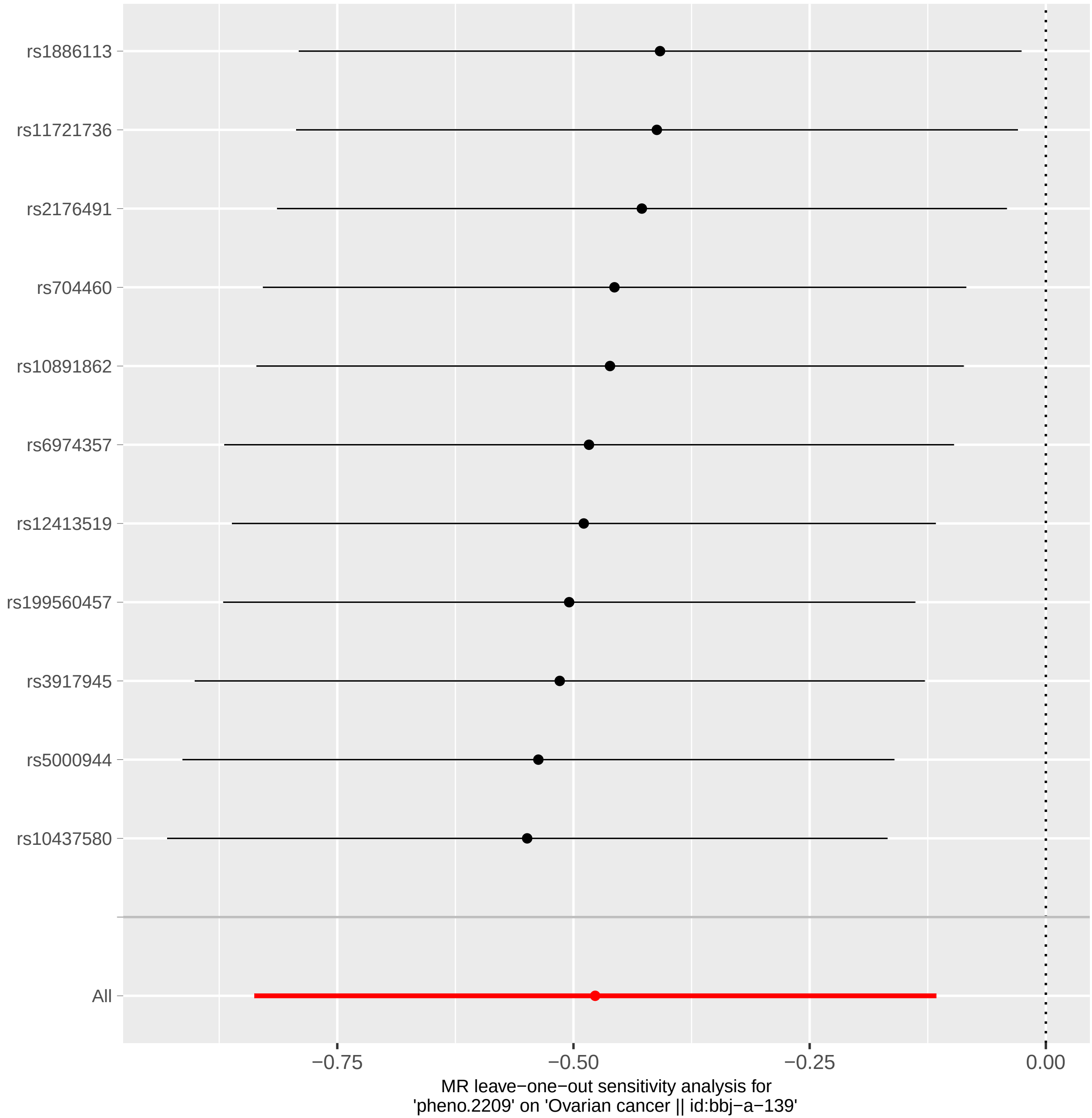
rs1886113
rs11721736
rs2176491
rs704460
rs10891862
rs6974357
rs12413519
rs199560457
rs3917945
rs5000944
rs10437580
All
−0.75
−0.50
−0.25
0.00
MR leave−one−out sensitivity analysis for
'pheno.2209' on 'Ovarian cancer || id:bbj−a−139'
